# Evaluation of inflammatory status in blood in patients with rosacea

**DOI:** 10.1038/s41598-023-36247-5

**Published:** 2023-06-05

**Authors:** Nermin Karaosmanoglu, Pınar Ozdemir Cetinkaya, Ozge Mine Orenay

**Affiliations:** 1 Dermatology and Venereology, Ankara Training and Research Hospital, Ankara, Turkey; 2 Dermatology and Venereology, Şişli Hamidiye Etfal Training and Research Hospital, Istanbul, Turkey

**Keywords:** Biomarkers, Diseases, Medical research

## Abstract

Rosacea is a chronic inflammatory skin disease that is characterized by recurrent episodes of erythema, telangiectasia and papulopustular lesions. Although the pathogenesis is not well established, growing knowledge suggests that multiple etiological factors play a role resulting in inflammation. The aim of the present study is to investigate inflammatory status of patients with rosacea by evaluating CBC parameters and systemic immune inflammation (SII) index and compare these parameters with control group. Thus, it is aimed to understand the role of systemic inflammation in the pathogenesis of the disease. This retrospective, case–control study included 100 patients with rosacea and 58 sex- and age-matched controls. Laboratory examinations including CBC, ESR, CRP, HDL, LDL, and triglyceride levels were recorded and neutrophil–lymphocyte ratio (NLR), monocyte-lymphocyte ratio (MLR), and platelet-lymphocyte ratio (PLR), monocyte-to-high-density lipoprotein ratio (MHR) and SII index were calculated. Monocyte and platelet count, SII index, ESR and CRP were significantly higher in patients with rosacea than in the control group. No statistically significant difference was found in other parameters. There was no significant correlation between disease severity and ESR, CRP, and SII index. The findings of this study suggest that apart from the inflammatory pathways at the level of skin, there is an inflammatory state in the blood of patients. Rosacea is a skin disease, but it may have systemic implications and/or associations that need to be clarified completely.

## Introduction

Rosacea is a chronic cutaneous syndrome that is characterized by recurrent episodes of erythema, telangiectasia and papulopustular lesions involving predominantly on the convexities of the central face (cheeks, chin, nose, and central part of forehead) of middle-aged women with skin phototypes I-III^1^.

The underlying pathophysiology is not well established, but growing knowledge suggests that there are multivariate set of pathogenic pathways including neurovascular, inflammatory, phymatous and ocular^1,2^. These pathways are also interconnected and according to their activity rate, they are responsible for the diversity of clinical manifestations. Neurovascular mechanisms are usually responsible for erythema, edema, burning sensation and reactivity of skin in rosacea^3^. Innate and adaptive immune activation (T helper 1 and T helper 17) along with mast cells and related biochemical mechanisms are responsible for mainly inflammatory features including erythema, telangiectasia, inflammation, pustule formation but also pruritus, pain, vascular responsivity^4,5^.

In the literature, there are numerous studies that reported significant associations between rosacea and cardiovascular diseases, hypertension, dyslipidemia, diabetes mellitus, metabolic syndrome, neurological, auto-immune, and psychiatric disorders along with an increased risk of cancer^6–8^. Moreover, there are various studies measuring biochemical and inflammatory markers such as C-reactive protein (CRP), erythrocyte sedimentation rate (ESR), uric acid, bilirubin and complete blood count (CBC) parameters such as mean platelet volume (MPV), neutrophil–lymphocyte ratio (NLR), monocyte-lymphocyte ratio (MLR), and platelet-lymphocyte ratio (PLR), monocyte-to-high-density lipoprotein ratio (MHR) in the blood of patients to evaluate inflammatory status of patients with rosacea^9–12^. Further studies are needed to elucidate whether there is a definite relationship between rosacea and chronic inflammatory systemic diseases and to understand the contribution of inflammatory markers in the blood sample of patients with rosacea to the pathogenesis of the disease.

In the present study, we aimed to investigate inflammatory status of patients with rosacea by evaluating CBC parameters such as MPV, NRL, MLR, PLR, MHR and systemic immune inflammation (SII) index, and compare these parameters with control group. With the current knowledge, it can be suggested that chronic inflammation predisposes to both rosacea and comorbidities. Thus, we aimed to investigate the role of systemic inflammation in the pathogenesis of the disease.

## Materials and methods

Between January 1 and December 31, 2020, a total of 100 patients with rosacea and 58 sex- and age matched controls who were admitted to the Department of Dermatology of Ankara Training and Research Hospital were included in the study. The institutional ethics committee of Ankara Training and Research Hospital approved the study (23/03/2022, E-93471371-514.99). All participants were informed about the study and a written consent form was obtained. The study was performed in accordance with the latest version of the "Helsinki Declaration" and "Guidelines for Good Clinical Practice.”

A retrospective chart review was carried out over a period of 1 year from January-December 2020 from the hospital’s electronic registration database. The diagnosis of rosacea was made clinically by the dermatologists who conducted the study. Subtypes of rosacea and clinical severity were also determined. Subtypes and severity of rosacea were determined according to standard grading system defined by the National Rosacea Society Expert Committee, in 2004. Primary (flushing, nontransient erythema, papules and pustules, telangiectasia) and secondary (burning or stinging, plaques, dry appearance, edema, ocular manifestations, peripheral location, phymatous changes) features of rosacea were graded and a global assessment of the disesase was done as mild, moderate and severe^13^. The demographic and clinical characteristics of the patient and control group, such as age and sex were recorded. Routine laboratory examinations including complete blood count, ESR, CRP, HDL (high density lipoprotein), LDL (low density lipoprotein), and triglyceride were also recorded and NLR, MLR, PLR, MHR, and SII index were calculated. SII index is calculated by the formula NLR x platelet.

Inclusion criteria the rosacea group were new diagnosis (without treatment) or a former diagnosis of rosacea who did not receive any systemic and/or topical drugs for the last 3 months that had the potential of changing the inflammatory status of blood by anti-inflammatory action. Exclusion criteria for the rosacea group were the consumption of drugs that may alter hematological, biochemical, and inflammatory parameters (such as iron, vitamin supplements, non-steroidal anti-inflammatory drugs). The control group was selected from volunteers of similar age and gender without any skin disease. Exclusion criteria for the control group were the presence of any facial dermatoses. Those who were diagonosed with cancer, severe infection, hematological diseases, heart failure, chronic kidney disease and who were pregnant or under 18 years of age were not included in either group.

### Statistical analysis

All analyses were carried out using the statistical package SPSS, Version 16.00 (Statistical Package for the Social Sciences, SPSS Inc., Chicago, IL, USA), and a p value less than 0.05 was considered statistically significant. The normality of the data was tested. Continuous variables are expressed as the mean ± standard deviation if they had a normal distribution or otherwise as the median and interquartile range. Categorical variables are expressed by number (percentage). Pearson’s Chi-square test were used for categorical variables. Independent samples were compared with Student’s t test and Mann–Whitney U test. Scale data were compared with Spearman's Rho correlation test. A post-hoc power analysis was conducted using G*Power Ver.3.0 Software® to test the difference between two independent group means using a two-tailed test, a medium effect size (d = 0.50), and an alpha of 0.05.

## Results

This case–control study included 100 patients with rosacea and 58 age- and sex-matched controls, and the power of this study was 0.85. The age and sex distributions of the study and control groups are presented in Table 1. Of the 100 patients with rosacea, 75 (75%) were female and 25 (25%) were male, and mean age of the study group was 49.79 ± 14.63 years. Fifty patients (50%) had erythematotelangiectatic subtype of rosacea, 39 patients (39%) had papulopustular subtype of rosacea, 7 patients (7%) had phymatous subtype of rosacea and 4 patients (4%) had ocular subtype of rosacea. Assessment of the clinical severity of the disease revealed that 52 patients (52%) had mild disease, 30 patients (30%) had moderate disease, and 18 patients (18%) had severe disease.

**Table 1:** Demographic features of the patient and control groups.

	Rosacea (*n* = 100)	Control (*n* = 58)	*p*
Sex (*n*/%)			
Female	75 (75%)	37 (63.8%)	0.135
Male	25 (25%)	21 (36.2%)	
Age (mean ± SD, years)	49.79 ± 14.63	46.49 ± 11.50	0.456

Data were expressed as mean ± SD and n (%) in categoric variables, respectively.

Student’s T test and Pearson's chi-square tests were used.

*SD* standard deviation.

Hematological, biochemical, and inflammatory parameters of the rosacea and control groups and their comparison are shown in Table 2. In patients with rosacea, the median monocyte was 0.53 (IQR:0.18) 10^9^/L, platelet was 276 (IQR:91) 10^9^/L, MPV was 10.5 (IQR:1.2) fl, ESR was 8 (IQR:10.5) mm/hour, CRP was 2.22 (IQR:4.34) mg/dl, and SII index was 517.20 (IQR:417.05) 10^9^/L. Monocyte and platelet count, MPV, SII index, ESR and CRP were statistically significantly higher in patients with rosacea than in the control group (p = 0.040, p = 0.006, p = 0.022, p = 0.026, p = 0.006, p < 0.001, respectively). No statistically significant difference was found in other parameters. SII index was evaluated according to clinical subtypes of rosacea and clinical rosacea severity. There was no statistically significant relationship between SII index and clinical subtypes of rosacea (*p* = 0.571) or between SII index and clinical rosacea severity (*p* = 0.485). There was no significant correlation between disease severity and MPV, monocyte count, ESR, CRP, and SII index (p = 0.012, p = 0.816, p = 0.71, p = 0.68, p = 0.611, respectively).

**Table 2:** Hematological, biochemical, and inflammatory parameters of patient and control groups and their comparison.

	Rosacea (*n* = 100)	Control (*n* = 58)	*p*
Hematological parameters			
Hb (mean ± SD, gr/dl)	13.64 ± 1.74	14.40 ± 1.48	0.003*
Neutrophil (median (IQR), 10^9^/L)	4.15 (1.2)	3.96 (2.03)	0.087
Lymphocyte (mean ± SD, 10^9^/L)	2.37 ± 0.70	2.19 ± 0.52	0.135
Monocyte (median (IQR), 10^9^/L)	0.53 (0.18)	0.50 (0.17)	0.040*
Platelet (median (IQR), 10^9^/L)	276 (91)	257 (82)	0.006*
MPV (median (IQR), fl)	10.5 (1.2)	10.1 (2.4)	0.022*
Biochemical parameters			
Triglyceride (median (IQR), mg/dl)	126 (101.5)	128 (74.25)	0.418
LDL (mean ± SD, mg/dl)	102.98 ± 3.83	110.89 ± 29.92	0.637
HDL (median (IQR), mg/dl)	47 (18.75)	48 (16)	
Inflammatory parameters			
NLR (median, IQR)	2.01 (1.09)	1.66 (0.76)	0.685
PLR (median, IQR)	124.9 (50.55)	108.25 (42.94)	0.224
MLR (median, IQR)	0.22 (0.11)	0.23 (0.08)	0.414
MHR (median, IQR, 10^8^/mg)	0.011 (0.006)	0.010 (0.006)	0.105
ESR (median, IQR, mm/h)	8 (10.5)	4 (7.5)	0.006*
CRP (median (IQR), mg/dl)	2.22 (4.34)	0.78 (2.38)	< 0.001*
SII index (median (IQR), 10^9^/L)	517.20 (417.05)	411.34 (216.80)	0.026*

Data are expressed as median (interquartile range) or mean ± standard deviation for those with nonparametric or parametric distribution, respectively. Independent samples were compared with Student’s t test and Mann–Whitney U test.

*SD* standard deviation, *IQR* interquartile range.

*p is statistically significant (*p* < 0.05).

## Discussion

Rosacea is a chronic inflammatory skin disease in which multiple etiological factors play a role including innate immune system, adaptive immune system, inflammasome, neurocutaneous mechanisms and genetic susceptibility^2^. Upregulation of keratinocyte-derived toll-like receptor 2 and proteinase-activated receptor 2, increased expression of cathelicidin and its bioactive form LL-37, activation of T helper type 1 and T helper 17 lymphocytes with their related immune mediators are mainly responsible for the inflammatory background of the disease^4,14,15^. It has also been proposed that both skin and gut microbiota contribute to the pathogenesis of rosacea^16,17^. Inflammatory pathways seem to play a key role in the pathogenesis of rosacea.

In clinical practice, the most frequently used indicators of inflammation in a patient are ESR, and CRP. However, besides these two well-known markers of inflammation, various new inflammatory values derived from CBC parameters have been identified. CBC is a routine and inexpensive laboratory test that is performed in almost every patient. It consists of the number of inflammatory cellular elements of the blood such as neutrophils, leukocytes, monocytes, and platelets which are non-specific cellular markers of overall inflammation. CBC derived parameters, NLR, PLR, MLR, and MHR are used as indicators of inflammation. A more recent parameter, SII index is a novel inflammation-based biomarker and is calculated by the formula NLR × platelet. Numerous studies were conducted to clarify use of hematological inflammatory parameters as a measure of inflammation, prognosis, and activity in many systemic chronic inflammatory diseases and cancers as well as dermatological diseases^18–23^. Recent studies reported elevated levels of hematological parameters and/or SII index in psoriasis, Behçet's disease, acne vulgaris (effect of systemic isotretinoin use), hidradenitis suppurativa and some of them also calculated a cut-off value for disease activation^19–23^.

In this study, we intended to evaluate the inflammatory status in blood of the patients with rosacea and measured CBC parameters, biochemical parameters, and inflammatory markers to elucidate the pathophysiological connection. To the best of our knowledge, this is the first study evaluating SII index in patients with rosacea along with CRP and ESR.

Chronic inflammatory process and comorbidities is an intriguing topic in rosacea pathogenesis and various studies were carried out to determine the role of oxidative stress, level of antioxidants, level of proinflammatory/inflammatory mediators and markers. In 2017, Akin Belli et al. investigated NRL, MHR, PLR, biochemical parameters, presence of metabolic syndrome and insulin resistance in patients with rosacea and they compared these findings with control group. They reported that NLR, LDL, total cholesterol, CRP, systolic and diastolic blood pressures, and presence of insulin resistance was significantly higher in patients with rosacea than in controls. They also found that MHR is significantly higher in patients with rosacea who had insulin resistance and metabolic syndrome and the MHC ratio was an independent predictor of metabolic syndrome in patients with rosacea^10^. In 2019, Altunisik et al. evaluated CBC parameters including MPV, RDW, NLR, MLR, and PLR in patients with rosacea and healthy controls (also in subgroups of demodex positive and demodex negative patients) and reported that there was no significant difference in these values between the patient and control group^9^. In our previous study in 2020, we evaluated serum uric acid (UA), CRP levels and presence of metabolic syndrome in patients with rosacea. Serum UA and CRP values were significantly higher in the rosacea group than values in the control group and metabolic syndrome was significantly more common in patients with rosacea whereas there was no significant correlation between serum UA level and clinical rosacea severity^11^. In 2020, Turkmen measured total bilirubin, direct bilirubin, indirect bilirubin, and uric acid values of patients with rosacea and compared these values with healthy controls. It was reported that significantly lower total bilirubin, direct bilirubin, indirect bilirubin, and uric acid values were detected in the patient group than in the control group^12^. In the present study, we evaluated the laboratory tests of 100 patients with rosacea and 58 control patients. Hallmark indicators of inflammation, ESR and CRP were significantly higher in patients with rosacea. Monocyte and platelet count, MPV and SII index were significantly higher in patients with rosacea while there was no significant difference neutrophil and lymphocyte count, NLR, PLR, MLR, MHR. We could not detect a significant correlation between disease severity and inflammatory status. Some results of our study were consistent with the studies in the literature but some of them were also contradictory. When all the results are interpreted together, it can be concluded that all CBC parameters do not seem to increase in rosacea, but the SII index calculated by a formula (NLR x platelet) from these parameters seems to better reflect the inflammatory state indicated by ESR and CRP. Finally, the detected inflammatory state does not appear to have any effect on disease severity.

Rosacea have been found to be associated with numerous systemic diseases including cardiovascular, neurological, gastrointestinal, metabolic/endocrine, allergic, rheumatological diseases and some cancers^6,8^. However, it is not completely understood whether the accompanying diseases occur in conjunction with rosacea or does rosacea cause these diseases? It is also not clear whether the comorbities proceed the onset of rosacea or vice versa. Moreover, further research is still needed to elucidate whether rosacea is a distinct disease of the skin or it is a disease with both skin and systemic implications. Holmes et al. evaluated the pathogenesis of rosacea and comorbid diseases in the perspective of genetic susceptibility, environmental factors and immune activation and they reported that they share common susceptibility alleles (histocompatibility complex/HLA), single nucleotide polymorphisms, and common innate, adaptive, and neurogenic inflammatory elements^8^. With these findings it can be suggested that chronic inflammation predisposes to both rosacea and systemic diseases so that it would be wise to evaluate the inflammatory status of patients with simple parameters and be suspicious about systemic diseases in those with higher values to achieve good clinical practice.

There are several limitations of our study. First of all, it is a retrospective case control study. Due to the retrospective nature of the study, we could not include detailed medical history and alcohol use and smoking habits of the patients. Rosacea is a disease with comorbidities and systemic diseases are important contributing factors to inflammmatory status of the body. Contribution of these factors to the inflammatory process in the blood could not be calculated. Second limitation is the moderate sample size. Further prospective studies with larger numbers of patients, constructed by considering confounders are needed to truly determine the inflammatory status of patients with rosacea. The main strength of the study is including the well-known inflammatory markers, ESR and CRP values. However, other important inflammatory molecules such as TNF-ɑ, IL-1, IL-6 levels were not evaluated in this study.

In conclusion, the findings in this study suggest that apart from the inflammatory pathways at the level of skin, there is an inflammatory state in the blood of patients. Rosacea is a skin disease, but it may have systemic implications and/or associations that need to be clarified completely. Further prospective studies, including larger number of patients are needed to determine whether SII index can be used to understand inflammatory state of patients with rosacea and complex relationship between the pathogenesis of rosacea and comorbidities.

## Data Availability

Datasets regarding this study would be available on request from the corresponding author.
